# *QuickStats:* Percentage* of Adults Aged ≥18 Years with Diagnosed Diabetes,^†^ by Urbanization Level^§^ and Age Group — National Health Interview Survey, United States, 2022^¶^

**DOI:** 10.15585/mmwr.mm7302a5

**Published:** 2024-01-18

**Figure:**
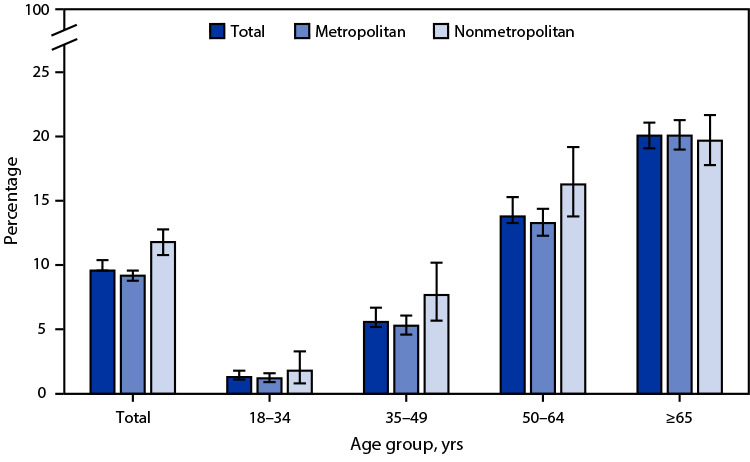
In 2022, 9.6% of adults aged ≥18 years had diagnosed diabetes, with the percentage lower among adults living in metropolitan areas (9.2%) compared with adults in nonmetropolitan areas (11.8%). The prevalence of diagnosed diabetes was lower in metropolitan areas only among those aged 35–49 years (5.3% versus 7.7%) and aged 50–64 years (13.3% versus 16.3%). The prevalence of diagnosed diabetes increased with age overall, from 1.3% among adults aged 18–34 years to 20.1% among adults aged ≥65 years, and in both metropolitan and nonmetropolitan areas.

**Source:** National Center for Health Statistics, National Health Interview Survey, 2022. https://www.cdc.gov/nchs/nhis.htm

